# Osteogenic potential of osteoblasts from neonatal rats born to mothers treated with caffeine throughout pregnancy

**DOI:** 10.1186/s12891-015-0467-8

**Published:** 2015-02-04

**Authors:** Amanda Maria Sena Reis, Lorena Gabriela Rocha Ribeiro, Natália de Melo Ocarino, Alfredo Miranda Goes, Rogéria Serakides

**Affiliations:** Núcleo de Células Tronco e Terapia Celular Animal (NCT-TCA) da Escola de Veterinária da Universidade Federal de Minas Gerais, Av. Antônio Carlos 6627, Caixa Postal 567, campus Pampulha da UFMG, CEP 30123-970, Belo Horizonte, MG Brazil; Laboratório de Imunologia Celular e Biologia Molecular do Departamento de Bioquímica e Imunologia, Instituto de Ciências Biológicas, Universidade Federal de Minas Gerais, Avenida Presidente Antônio Carlos, 6627, CEP: 30.161-970, Belo Horizonte, Minas Gerais Brazil

**Keywords:** Osteoblasts, Caffeine, Neonate, Osteogenic differentiation, Rat

## Abstract

**Background:**

Caffeine is an active alkaloid that can cause damage to bones in formation during prenatal life into adulthood. This compound can pass across the placenta and into the mother’s milk, causing a reduction in bone formation, growth and mass. The objective of this study was to examine the osteogenic potential of osteoblasts extracted from neonatal rats born to mothers treated with caffeine throughout pregnancy.

**Methods:**

Twenty-four adult Wistar rats were randomly divided into four groups, consisting of one control group and three groups that were treated with 25, 50, or 100 mg/kg of caffeine by an oral-gastric probe throughout the duration of the experimental period (pregnancy). At birth, three puppies from each dam in each group were euthanized, and osteoblasts were extracted from the calvaria of these pups for *in vitro* testing.

**Results:**

The osteoblasts extracted from the pups of rats that received 50 mg/kg caffeine during pregnancy exhibited increased expression of osteocalcin, osteopontin, sialoprotein, runx-2, alkaline phosphatase and type I collagen transcripts, resulting in increased synthesis of mineralization nodules.

**Conclusions:**

Neonates from rats treated with 50 mg/kg caffeine during pregnancy contained osteoblasts with a higher osteogenic potential characterized by increased expression of osteocalcin, osteopontin, sialoprotein, runx-2, alkaline phosphatase and type I collagen and increased synthesis of mineralization nodules.

## Background

Caffeine is a pharmacologically active xanthine (1,3,7-trimethylxanthine) that is present in coffees, teas, soft drinks, food, and drugs [1].

Despite the beneficial effects of caffeine on the central nervous system and the respiratory system, characterized by reduced fatigue and bronchodilation, respectively, the adverse effects of caffeine on the body are more widely documented. Caffeine is known to cause insomnia and anxiety as well as increased blood pressure and pulse. In addition, caffeine is considered a risk factor for osteoporosis and periodontal disease and a causative agent of fetal malformations, reductions in body weight, reduced length of long bones and reduced length of vertebrae in newborns [1-7].

Caffeine crosses the placenta leading to substantial amounts in amniotic fluid, umbilical cord blood, newborn plasma and newborn urine and is also carried by the mother’s milk in both mice and humans [8-11]. Pregnant women in the third trimester of pregnancy with low caffeine consumption had mean serum levels of caffeine in their umbilical cord blood of 0.48 μg/ml (0 to 10.49 μg/ml). Pregnant women with caffeine intake greater than 300 mg/day averaged 2.1 μg/ml of caffeine in their cord blood [9]. Even in humans, maternal ingestion of 36–335 mg of caffeine has been shown to be transmitted in the milk, which contained between 2.09 to 7.17 μg/ml caffeine [11]. Previous studies have shown that caffeine causes changes in the bones of fetuses and young rats. In rat fetuses born to mothers treated with high doses of caffeine, teratogenic changes such as cleft palate, limb malformations, and ectrodactyly [12-14] have been observed, as well as reduced bone mass [15] and reduced bone mineral content [4]. In the offspring of rats treated with caffeine at doses of 25, 50 and 100 mg/kg, osteopenia in the long bones and vertebrae was observed [15]. High doses of caffeine inhibit the endochondral bone growth of offspring when administered to rats during pregnancy and lactation. The effects of caffeine are more damaging to cartilage growth in newborns than to pups at weaning [15]. In humans, caffeine intake above 540 mg/day during pregnancy is associated with decreased weight and fetal growth, and combining caffeine intake with smoking and alcohol consumption may further increase risk [16,17].

The mechanism that underlies the changes caused by caffeine, especially in the bones, requires further study. The effects of caffeine on bone metabolism are controversial. Some studies suggest that the consumption of caffeine has been associated with low bone mass and increased fracture risk and that caffeine directly enhances differentiation and maturation of osteoclasts [18,19].

Additionally, it has been postulated that one mechanism by which caffeine alters bone formation and growth is by affecting mesenchymal stem cells and osteoblasts [20,21]. *In vitro* studies have concluded that caffeine can potentially harm osteoblasts, causing a reduction in viability and synthetic activity [20-23]. The dosage of caffeine can influence the osteogenic differentiation of mesenchymal stem cells [24]. Low doses of caffeine (0.1 mM) in culture medium can increase the mineralization, alkaline phosphatase activity and differentiation genes such as osteocalcin, osteoprotegerin, and Runx-2, whereas higher doses (≥0.3 mM) can suppress differentiation [24]. However, little is known about the effects, associated factors and molecular mechanisms of caffeine action during skeletogenesis. Most previous studies that have addressed this issue have investigated the effects of adding caffeine to cultured mesenchymal stem cells and osteoblasts [20-23]. This, however, is the first study to examine the osteogenic potential of osteoblasts extracted from neonatal rats born to mothers treated with caffeine throughout pregnancy.

The osteogenic potential of the cells was evaluated using parameters such as alkaline phosphatase activity; collagen synthesis; gene expression of Runx-2, collagenous proteins and non-collagenous proteins (osteocalcin, osteopontin, sialoprotein); and the synthesis and mineralization of the extracellular matrix. Runx-2 regulates collagenous and non-collagenous protein factors that play a key role in the various stages of differentiation in the extracellular matrix [25-29], while alkaline phosphatase is an early marker of osteogenic differentiation [30,31]; these factors are thus important to evaluate.

Therefore, with the aim of elucidating the mechanism of action of caffeine in osteoblasts, this study analyzed the effects of caffeine on the synthetic activity of cells extracted from the calvaria of newborn rats whose mothers received different doses of caffeine during pregnancy.

## Methods

### Animals and study design

Twenty-four two-month-old female Wistar rats were used in this study. The animals were housed six per cage by experimental group in a 12-h light/dark cycle. They were fed commercial rat chow containing 22% crude protein, 1.4% calcium and 0.6% phosphorus. Food and water were provided ad libitum to all animals. All experimental procedures were approved by the Institutional Ethics Committee in Animal Experimentation at *Universidade Federal de Minas Gerais* (UFMG) (protocol no. 177/2010).

After a 30-day adaptation period, the rats were randomly divided into four groups, consisting of three groups that were treated with different doses of caffeine and a control group. The treated groups (6 animals/group) received daily caffeine (Sigma Aldrich, St. Louis, MO, USA) diluted in 5 mL of distilled water at doses of 25, 50 or 100 mg/Kg. The doses of caffeine used in this study were chosen based on previously observed effects on endochondral ossification in the offspring of rats treated with caffeine [15].

The doses were administered by an oral-gastric probe at the same time each day over the course of the experimental period. As a placebo, the control group received 5 mL of distilled water daily by an oral-gastric probe. The females were subjected to vaginal cytology to monitor the estrous cycle [32]. The female rats that were in estrus and proestrus were kept in plastic cages with adult male rats for 12 h. After this period, vaginal smears were obtained daily to detect spermatozoa. Copulation was confirmed by the presence of spermatozoa in the vaginal cytology samples, and the day of copulation was considered Day 0 of gestation. After copulation, the females were kept individually in plastic cages. The animals in the treated and control groups continued to receive caffeine or water, respectively, by an oral-gastric probe throughout the duration of the experimental period (pregnancy).

At birth, three pups from each dam in each experimental group were euthanized, and osteoblasts were extracted from the calvaria of these pups. The cells were pooled to obtain a cell suspension for in vitro tests. The means and standard deviations were determined from six replicates for each experimental group.

#### Harvest and culture of osteoblasts from calvaria of newborn pups

Calvarial osteoblast cultures were established as described previously [26,33]. Briefly, 2 day-old neonatal Wistar rats were removed from their cages, and their heads were cut off and immersed in phosphate-buffered saline (PBS) (Synth, São Paulo, SP, Brazil). The calvaria were then dissected out and processed for cell isolation and culture. The calvaria were fragmented and incubated for 15 min with 1% trypsin (Gibco, Grand Island, NY, USA) and then for 60 min at 37°C in PBS with 0.25% collagenase (Type I; Sigma, St Louis, MO, USA). The cells that dissociated from the fragments of calvaria were washed several times in PBS, suspended in DMEM medium (Gibco, Grand Island, NY, USA) supplemented with 10% fetal bovine serum (Gibco, Grand Island, NY, USA) and antibiotics (60 μg/L gentamicin, 25 μg/L amphotericin B, 100 U/mL penicillin and 100 μg/mL streptomycin; Gibco, Grand Island, NY, USA), and collected in a 75-cm^2^ culture flask containing 10 mL of culture medium. The cells were grown at 37°C in a 5% CO_2_ atmosphere for 3 days. The non-adherent cell population was removed, and the adherent layer was washed once with fresh medium. The culture medium was changed twice a week during culturing.

#### Culture of osteoblasts in osteogenic differentiation medium

After the fourth passage, the adherent cells were harvested by treatment with trypsin. They were then counted and plated in 6- and 24-well culture plates (1 × 10^4^ cells/cm^2^). The cells were cultured in osteogenic medium, which consists of DMEM (Gibco, Grand Island, NY, USA) supplemented with 10% fetal bovine serum (Gibco, Grand Island, NY, USA), antibiotics (60 μg/L gentamicin, 25 μg/L amphotericin B, 100 U/mL penicillin, 100 μg/mL streptomycin; Gibco, Grand Island, NY, USA), 10 mM *β*-glycerophosphate (Sigma, St. Louis, MO, USA), 50 μg/mL ascorbic acid (Merck, Germany) and 10 nM dexamethasone (Sigma-Aldrich, St Louis, MO, USA) [26,33]. The cells were grown at 37°C in a 5% CO_2_ atmosphere. The cells were grown for 7, 14 and 21 days to assess the conversion of MTT into formazan crystals, collagen synthesis, alkaline phosphatase activity, production of mineralized nodules and the expression of osteocalcin, osteopontin, bone sialoprotein, alkaline phosphatase and type I collagen via qRT-PCR.

#### MTT reduction assay

MTT conversion is a measure of cell viability. Briefly, the cells were incubated with MTT (3-(4,5-dimethylthiazol-2-yl)-2,5 diphenyltetrazolium bromide) (Invitrogen, Eugene, Oregon, USA) for 2 h at 37°C. After incubation with MTT, the cells were treated for 12 h with a solubilization solution (sodium dodecyl sulfate in 10% HCl) (Synth, São Paulo, SP, Brazil), and the absorbance of the solubilized MTT formazan product was measured at 595 nm using a microtiter plate reader [23,24]. The means and standard deviations were determined from six replicates for each experimental group.

#### Alkaline phosphatase activity

At the end of each period (7, 14 and 21 days), the osteoblasts from the newborn pups of all groups were cultured in 24-well plates and washed with 0.15 M PBS. Two hundred microliters of BCIP/NBT solution (1 mL of alkaline phosphatase buffer, 4.4 μL of NBT {nitro-blue tetrazolium chloride} and 3.3 μL of BCIP {5-bromo-4-chloro-3’-indolylphosphate p-toluidine salt}) (Invitrogen, Camarilho, CA, USA) were added to each well. The plates were incubated for 2 h at 37°C. After incubation with BCIP/NBT, the cells were treated with a solubilization solution (sodium dodecyl sulfate in 10% HCl) (Synth, São Paulo, SP, Brazil), and the absorbance of the solubilized BCIP/NBT was measured at 595 nm using a microtiter plate reader [34,35]. The means and standard deviations were determined from six replicates for each experimental group.

#### Collagen synthesis

At the end of each period (7, 14 and 21 days), the osteoblasts of newborn pups of all groups were cultured in 6-well plates and washed with 0.15 M PBS (Synth, São Paulo, SP, Brazil). Four milliliters of Bouin solution (Merk, Germany) was added to each well. The samples were incubated for 2 h at 37°C and subsequently moved to a refrigerator. The following day, the cells were washed, dried and incubated in Sirius Red (Reagent, Rio de Janeiro, RJ, Brazil) (2 mL) for 30 min. After the dye was removed, the cells were washed with HCl (Synth, São Paulo, SP, Brazil) and NaOH (Synth, São Paulo, SP, Brazil) and incubated for 30 min. Subsequently, 100 μL from each well was transferred to a 96-well plate, and the absorbance of each sample at 540 nm was analyzed using a plate reader [33]. The means and standard deviations were determined from six replicates for each experimental group.

#### Mineralization assay

The cells were cultured in six-well culture plates with 22 × 22 mm coverslips. The cells were washed in PBS (Synth, São Paulo, SP, Brazil), fixed in 4% paraformaldehyde (Synth, São Paulo, SP, Brazil) and rinsed with deionized water. After the addition of a 5% silver nitrate solution (Synth, São Paulo, SP, Brazil), the wells were exposed to light for 2 h. The plates were rinsed with deionized water, and the residual silver nitrate was neutralized by 5% sodium thiosulfate (Synth, São Paulo, SP, Brazil); after neutralization, the calcium deposits were visible by dark brown staining. The percentage of mineralized nodules was assessed by light microscopy. The mean percentage of nodules was quantified at 21 days of osteogenic differentiation; 50 fields were counted using a 10× objective and a 121-point graticule superimposed on the histological sections [36]. The means and standard deviations were determined from six replicates for each experimental group.

#### Real-time quantitative PCR (qRT-PCR)

Total mRNA was extracted using TRIzol reagent (Ambion, Carlsbad, CA, USA) according to the manufacturer’s instructions. Osteoblasts from each experimental group cultured in osteogenic medium for 7, 14, or 21 days were assayed for osteocalcin, osteopontin, bone sialoprotein, alkaline phosphatase and type I collagen expression. Osteoblasts cultured in osteogenic medium for 21 days were assayed for runx-2 expression. The means and standard deviations were determined from four replicates for each experimental group. One microgram of RNA was subjected to cDNA synthesis using a SuperScript III Platinum Two-Step qPCR kit with SYBR Green (Invitrogen, Carlsbad, CA, USA). The qRT-PCR reactions were conducted in a Smart Cycler II thermocycler (Cepheid, Carpinteria, CA, USA). The one-step qRT-PCR amplification started with reverse transcription for 120 s at 50°C, followed by PCR with the following parameters: 45 cycles of 15 s at 95°C and 30 s at 60°C. At the end of each run, the fluorescence data were analyzed to obtain CT values. Gene expression was calculated using the 2^-ΔΔCt^ method, where the values from samples were averaged and normalized to GAPDH CT values. The specific primers used are shown in Table 1. The results for each group were compared quantitatively after normalization to the expression of *Rattus norvegicus* GAPDH.

**Table 1:** **List of genes and oligonucleotide sequences of the primers used for real-time RT-PCR**

**Gene**	**Primers: 5′-3′**	**Access number**
GAPDH	*Foward*: CAACTCCCTCAAGATTGTCAGCAA	NM 002046
	*Reverse*: GGCATGGACTGTGGTCATGA	
Type 1 collagen	*Foward*: ACGTCCTGGTGAAGTTGGTC	NM 000088
	*Reverse*: CAGGGAAGCCTCTTTCTCCT	
Osteocalcin	*Foward*: CATCTATGGCACCACCGTTT	NM 013414.1
	*Reverse*: AGAGAGAGGGAACAGGGAGG	
Bone sialoprotein	*Foward*: TGTCCTTCTGAACGGGTTTC	NM 012587.2
	*Reverse*: CTTCCCCATACTCAACCGTG	
Osteopontin	*Foward*: ATCTCACCATTCCGATGAATCT	AB001382
	*Reverse*: TCAGTCCATAAGCCAAGCTATCA	
Alkaline Phosphatase	*Foward*: CTAGTTCCTGGGAGATGGTA	AC_000073.1
	*Reverse*: GTGTTGTACGTCTTGGAGAGA	
Runx-2	*Forward*: GCGTCAACACCATCATTCTG	NM_004348
	*Reverse:* CAGACCAGCAGCACTCCATC	

### Statistical analysis

The means and standard deviations were determined for each variable. We performed ANOVA and compared the means using an SNK test. Differences were considered to be significant at p < 0.05 [37].

## Results

### Conversion of MTT to formazan crystals

**Figure 1:**
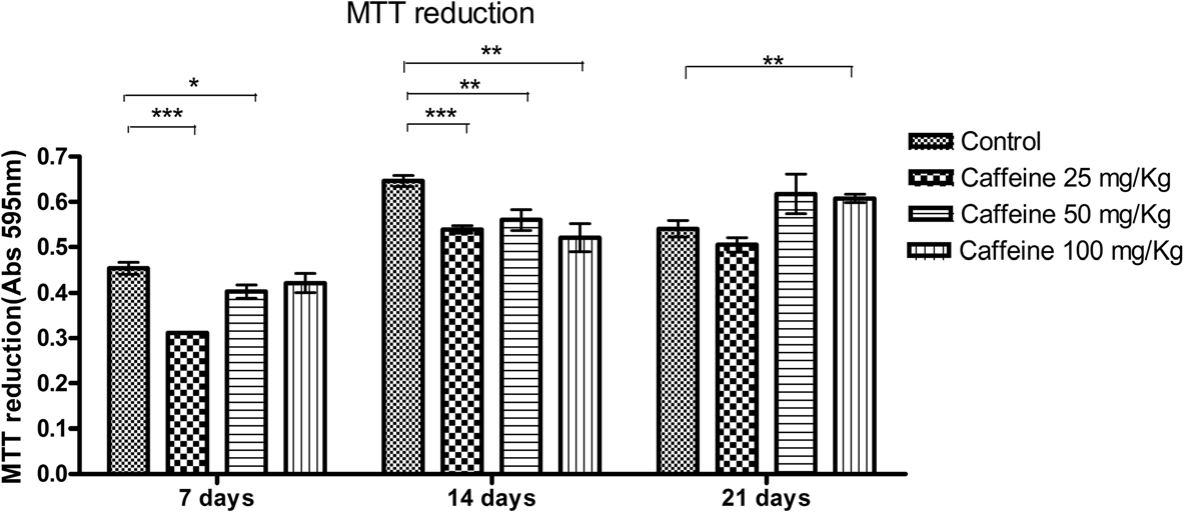
**Cell viability (mean ± standard deviation) characterized by conversion of MTT into formazan crystals by calvarial osteoblasts taken from newborn control rats or those exposed to various concentrations of caffeine (25, 50, 100 mg/Kg) during pregnancy and subsequently cultured in osteogenic differentiation medium for 7, 14 or 21 days.** *p <0.05, **p < 0.01, ***p < 0.001.

The conversion of MTT into formazan crystals varied with the dose of caffeine administered to the mother and the culture period. At 7 days, the groups treated with 25 and 50 mg/kg caffeine showed less conversion of MTT into formazan crystals than the control group. However, at 14 days, osteoblasts from all the caffeine-treated groups (25, 50 and 100 mg/kg) showed decreased conversion of MTT into formazan crystals compared with the controls (p ≤ 0.05). At 21 days, the groups treated with 25 and 50 mg/kg caffeine showed no difference in the conversion of MTT to formazan crystals relative to the control group; however, the 100 mg/kg group showed a significant increase compared with the control group (Figure 1).

### Alkaline phosphatase activity and synthesis of collagen

**Figure 2:**
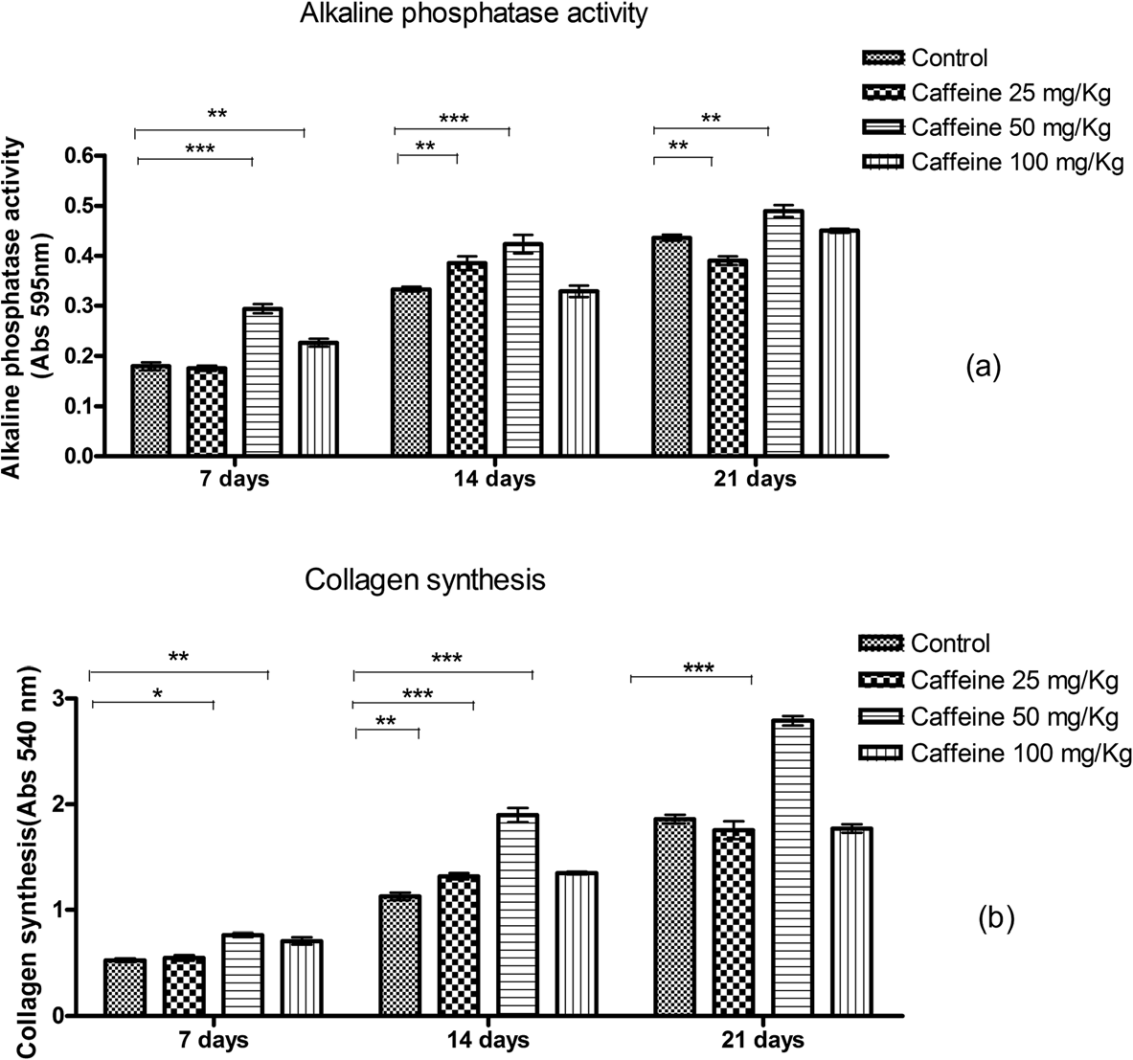
**Alkaline phosphatase activity (a) and synthesis of collagen (b) (mean ± standard deviation) in calvarial osteoblasts taken from newborn control rats or those exposed to various concentrations of caffeine (25, 50, 100 mg/Kg) during pregnancy and subsequently cultured in osteogenic differentiation medium for 7, 14 or 21 days.** *p < 0.05, **p < 0.01, ***p < 0.001.

At 7 days of culture, the groups treated with 50 and 100 mg/kg caffeine showed significantly higher alkaline phosphatase activity than the controls. At 14 days, the osteoblasts isolated from the 25 and 50 mg/kg groups showed significantly higher alkaline phosphatase activity than the control group. At 21 days of culture, only the group treated with 50 mg/kg caffeine maintained significantly higher alkaline phosphatase activity over than of the control group (Figure 2a).

At 7 days of culture, the groups treated with 50 and 100 mg/kg caffeine showed significantly higher collagen synthesis than the control group. At 14 days of culture, all three caffeine-treated groups showed significantly higher collagen synthesis than the controls. However, at 21 days of culture, only the osteoblasts cultured from the 50 mg/kg group maintained this increased collagen synthesis (p ≤ 0.05) (Figure 2b).

### Synthesis of mineralization nodules

**Figure 3:**
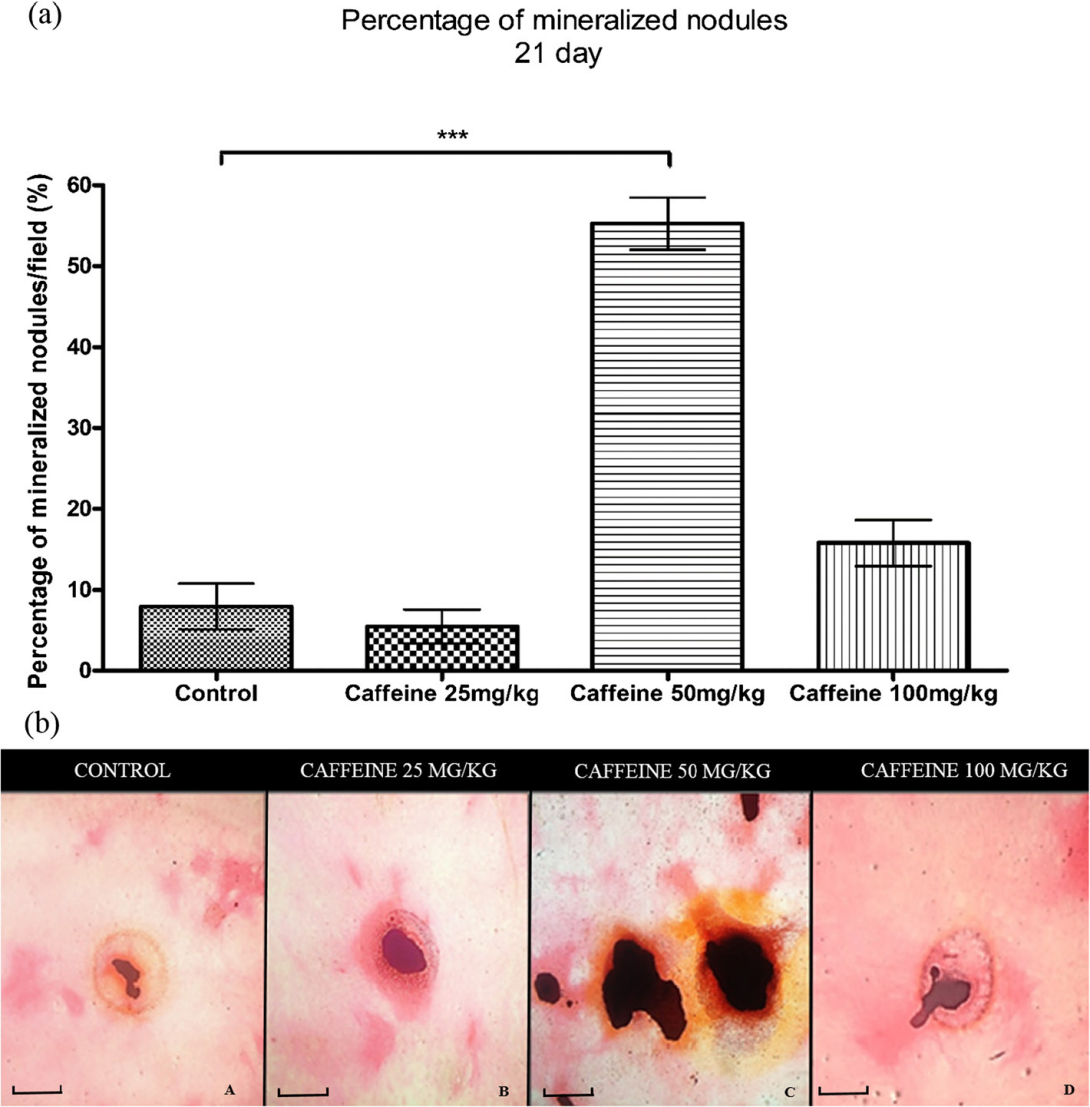
**Percentage of mineralization nodules/field (mean ± standard deviation) (a) and Von Kossa staining (b) of calvarial osteoblasts taken from newborn control rats and those exposed to various concentrations of caffeine (25, 50, 100 mg/Kg) during pregnancy and subsequently cultured in osteogenic differentiation medium for 21 days.** ***p < 0.001. Bar = 58.5 μm.

At 7 and 14 days of culture, there was variation among the cultures in the percentages of mineralization nodules, but there were no significant differences between the groups. However, at 21 days, the amount of variation was reduced, as indicated by a decrease in the standard deviations. The osteoblast cultures from the 50 mg/kg caffeine-treated group showed a significant increase in the percentage of mineralization nodules compared with the control group (Figure 3a). Furthermore, the calcium content of the nodules, as reflected by black staining, was higher in the group treated with 50 mg/kg caffeine than in the other groups (Figure 3b).

### Expression of osteogenic genes

**Figure 4:**
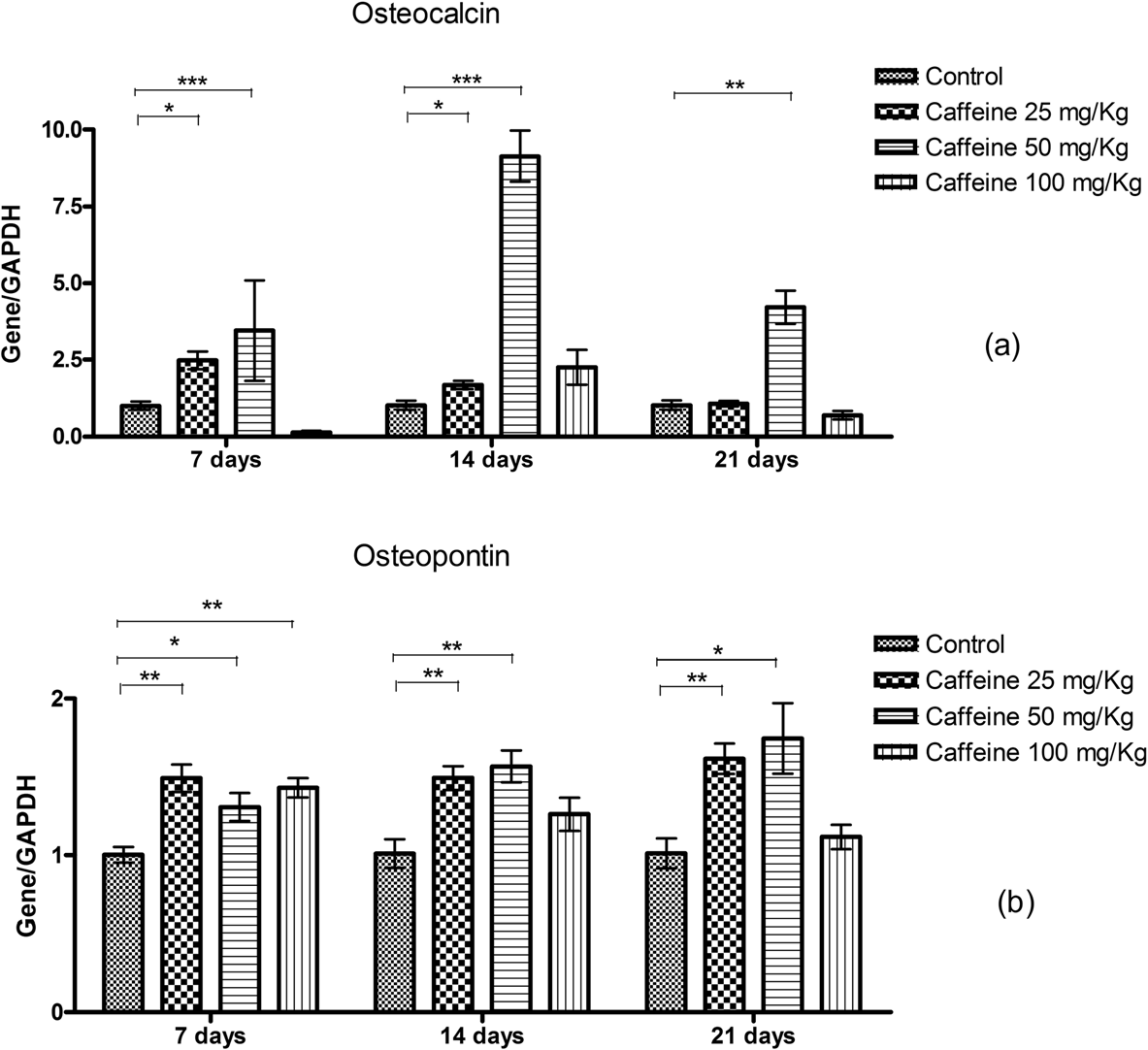
**The relative quantification (mean ± standard deviation) of osteocalcin (a) and osteopontin (b) transcripts as measured by real-time RT-PCR in calvarial osteoblasts taken from newborn control rats and those exposed to various concentrations of caffeine during pregnancy and subsequently cultured in osteogenic differentiation medium for 7, 14 or 21 days.** *p <0.05, **p < 0.01, ***p < 0.001.

**Figure 5:**
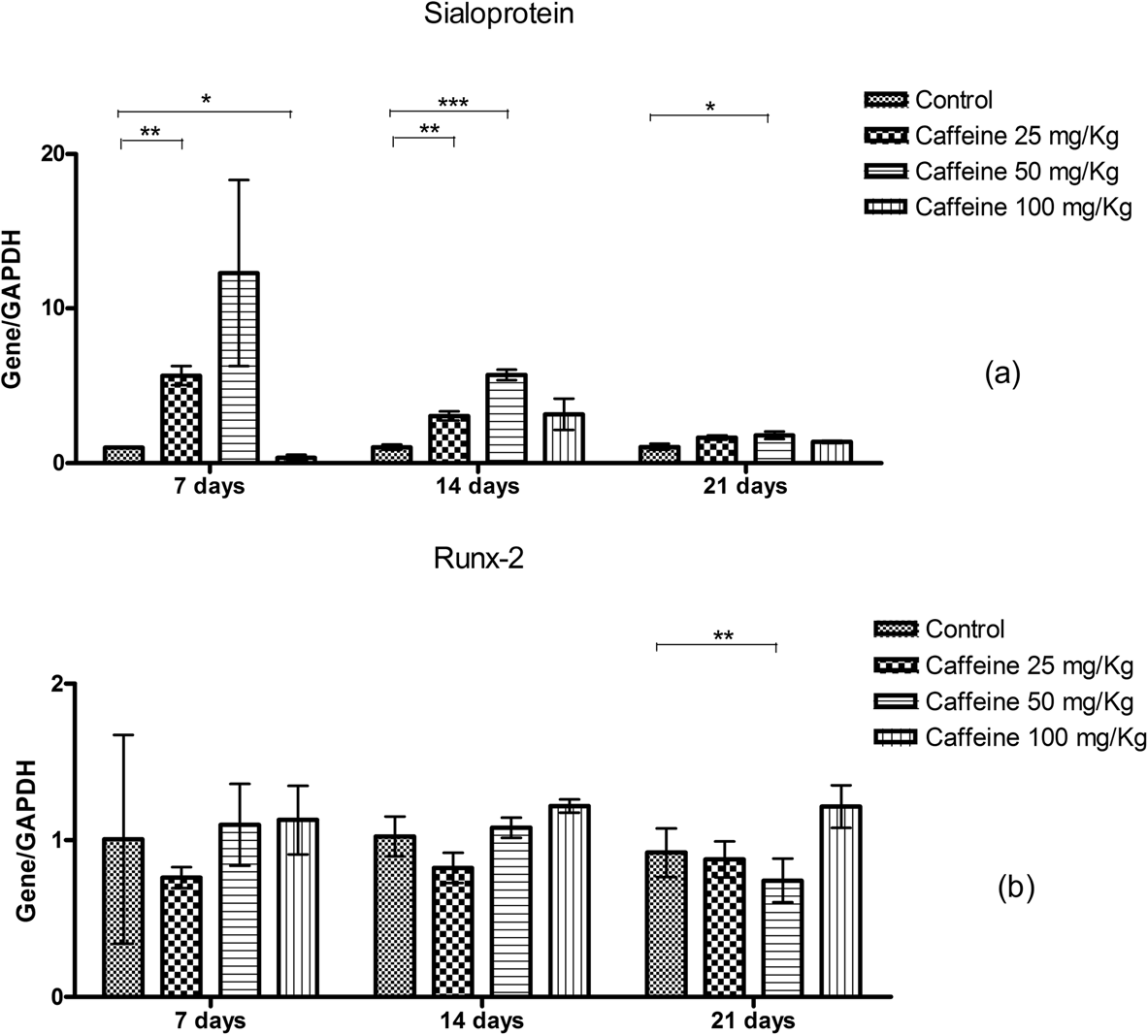
**The relative quantification (mean ± standard deviation) of sialoprotein (a) and runx-2 (b) transcripts as measured by real-time RT-PCR in calvarial osteoblasts taken from newborn control rats and those exposed to various concentrations of caffeine (25, 50, 100 mg/Kg) during pregnancy and subsequently cultured in osteogenic differentiation medium for 7, 14 or 21 days.** *p <0.05, **p < 0.01, ***p < 0.001.

At 7 days, the group treated with 25 mg/kg caffeine showed a significant increase in osteocalcin expression, while the 100 mg/kg group showed a significant reduction in the expression of this gene. However, at 14 and 21 days of culture, the osteoblasts of the 50 mg/kg caffeine-treated group showed a significant increase in the expression of osteocalcin compared with the control group (Figure 4a). Osteopontin expression was significant higher in all of the caffeine-treated groups (25, 50 and 100 mg/kg) at 7 days of culture than in the control group. However, at 14 and 21 days of culture, only the osteoblasts from the 25 and 50 mg/kg groups showed significantly higher expression of osteopontin (Figure 4b). At 7 days, sialoprotein expression was significantly increased in the 25 mg/kg group, but significantly decreased in the 100 mg/kg group. At 14 days, osteoblasts from the groups treated with 25 and 50 mg/kg caffeine showed significantly higher expression of sialoprotein than the control group. However, at 21 days, only osteoblasts from the group treated with 50 mg/kg caffeine maintained significantly higher sialoprotein expression than the controls (Figure 5a).

At 21 days of culture, only the group treated with 50 mg/kg caffeine showed a significant increase in runx-2 expression compared with controls (Figure 5b). Cells from all other experimental periods and doses exhibited no significant differences in runx-2 expression compared with the control group.

**Figure 6:**
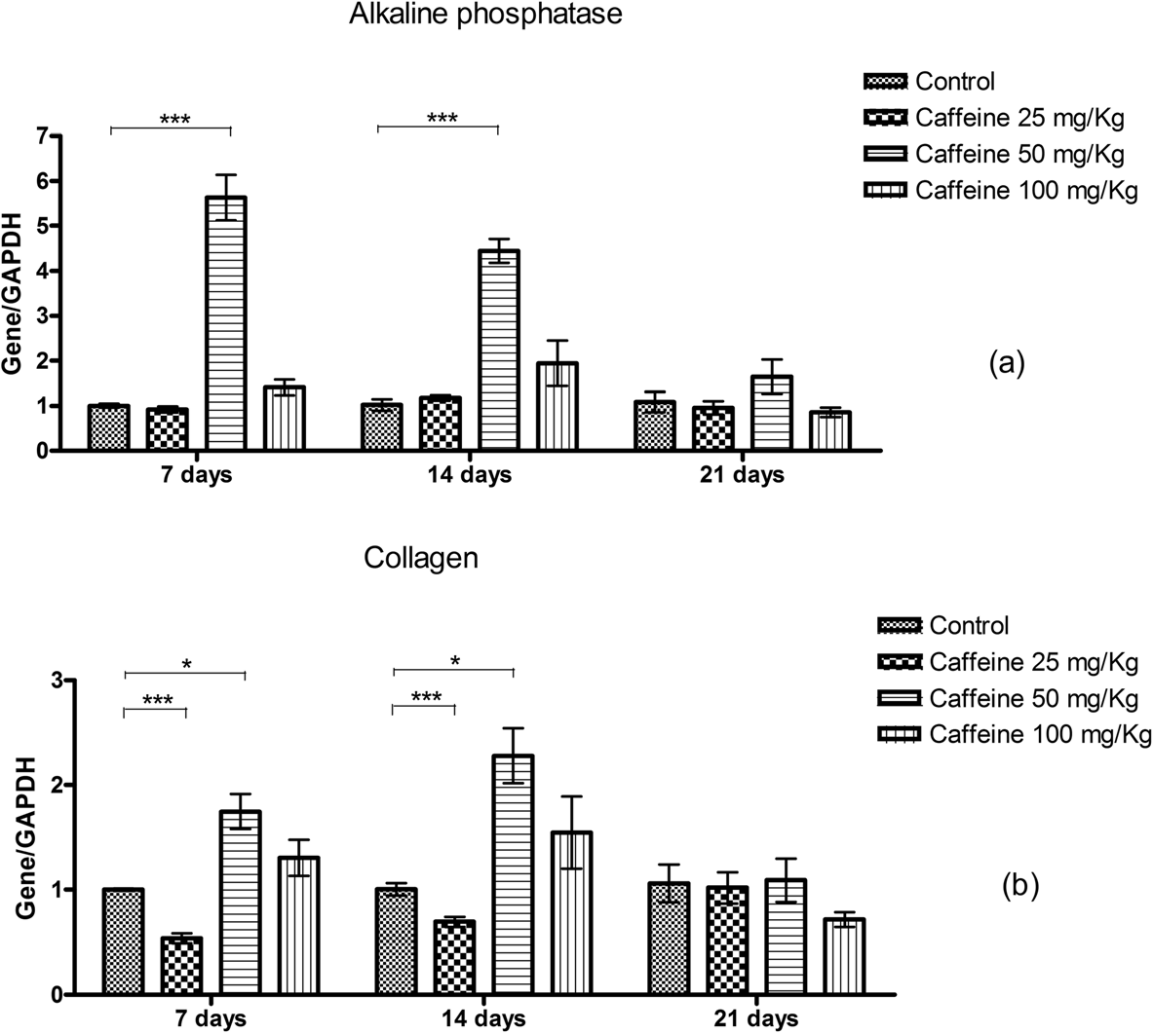
**The relative quantification (mean ± standard deviation) of alkaline phosphatase (a) and type I collagen transcripts (b) as measured by real-time RT-PCR in calvarial osteoblasts from newborn control rats and those exposed to various concentrations of caffeine (25, 50, 100 mg/Kg) during pregnancy and subsequently cultured in osteogenic differentiation medium for 7, 14 or 21 days.** *p <0.05, **p < 0.01, ***p < 0.001.

At 7 and 14 days of culture, alkaline phosphatase expression was significantly increased compared with controls only in the group treated with 50 mg/kg caffeine. At 21 days, there were no differences in alkaline phosphatase expression between the groups (Figure 6a). At 7 and 14 days, the group treated with 25 mg/kg caffeine had lower type I collagen expression than the controls, and the group treated with 50 mg/kg caffeine had higher expression. At 21 days of culture, there were no significant differences in type I collagen expression between the groups (Figure 6b).

## Discussion

Previous studies with cultured osteoblasts showed that caffeine can be harmful [20-23] and may be considered a risk factor for osteoporosis [18,29] and a causative agent of fetal malformations [5,12,14,15]. The mechanisms that underlie these changes in bone tissue remain poorly understood [5,12,15,38-40]. To the best of our knowledge, this work describes for the first time the effects of caffeine administration during pregnancy on the osteogenic potential of osteoblasts derived from pups.

The majority of cells extracted from calvaria are osteoblasts, although the derived cell population also includes pre-osteoblasts [41]. In the present study, these cells were evaluated after passaging three times in osteogenic medium to stimulate the differentiation of all extracted cells. To characterize and compare the osteogenic potential of the osteoblasts extracted from each experimental group, several parameters were evaluated: alkaline phosphatase activity, collagen synthesis, and the synthesis and mineralization of the extracellular matrix. During the process of cell differentiation, osteoblasts produce the bone extracellular matrix, which is composed of collagenous and non-collagenous proteins. Many of these proteins play a key role in the various stages of differentiation and mineralization of the bone matrix; here we highlight osteocalcin, sialoprotein and osteopontin [20,41,42], which were evaluated in this study.

Our results were surprising and interesting. In contrast with previously reported results from *in vitro* studies [20,21], we found that caffeine, predominantly at 50 mg/kg, increased the osteogenic potential of osteoblasts, as characterized by increased alkaline phosphatase activity, collagen synthesis, mineralization of nodules and expression of osteogenic genes including osteocalcin, osteopontin, Runx-2, sialoprotein, alkaline phosphatase and type I collagen.

The methodology used and the results obtained in this study were different from those reported by other researchers; previous studies showed that the viability and activity of osteoblasts decreased significantly when exposed to increasing doses of caffeine (0.4, 0, 5, 1.0 and 10 mM) in the culture medium [20-23]. The most important aspect of our method of caffeine administration is that the drug was not added to the culture medium as in most of the previous studies; we administered caffeine to mothers during pregnancy, and the drug passed through the placenta to the fetus. The doses of caffeine used in this study were chosen based on previously observed effects on endochondral ossification in the offspring of rats treated with caffeine [15]. When added directly to the culture medium, caffeine inactivates cell survival signaling and promotes programmed cell death by a mitochondria-dependent cascade; cell death thus occurs by apoptosis and necrosis [20-23]. Furthermore, there is decreased expression of genes, enzymes and proteins expressed during osteogenesis, such as Runx-2, alkaline phosphatase, type I collagen, osteocalcin, osteopontin, and histones [21-23,43]. The results obtained with the addition of caffeine to osteoblast cultures demonstrate that in addition to reducing cell viability, caffeine also inhibits the synthesis of the extracellular matrix [22,23].

However, *in vitro* assays may be different from *in vivo* and *ex vivo* assays because the cellular microenvironment of the organism is difficult to reproduce *in vitro* due to numerous interdependent intrinsic and extrinsic factors. The control of proliferation, differentiation and cell maintenance is carried out by genes, cytokines, developmental and growth factors and cellular interactions [44-46]. Thus, even with our knowledge of the regulation of the cellular microenvironment, it is difficult to develop *in vitro* models that can simulate drug effects in the body [44-46]. Although *in vitro* assays with cultured osteoblasts reproduce the cellular sequences that occur before and during the formation of bone matrix *in vivo* [41], the drugs used in in vitro studies are added directly to the culture medium and not metabolized by the body. This lack of drug metabolism may potentiate the effects of drugs on cells [21,43]. The aim of this study was to observe the effects of caffeine on the metabolism of osteoblasts in pups whose mothers were exposed to caffeine during pregnancy, taking into consideration the metabolism of the drug by the mother and its passage to the fetus through the placenta.

At 7 and 14 days, alkaline phosphatase activity and collagen synthesis were significantly higher in the group that received 50 mg/kg caffeine than in controls, based on both colorimetric assays and RT-PCR. Alkaline phosphatase is an early marker of osteogenic differentiation [47,48]; therefore, the increase in alkaline phosphatase suggests an increase in differentiation and early osteogenesis in the treated group compared with the control group. However, the increase in the gene transcript does not necessarily mean that the protein was also increased. In tests of the conversion of MTT into formazan crystals, osteoblasts from rat neonates that received a dose of 50 mg/kg caffeine showed a lower conversion of MTT into formazan at 7 and 14 days of culture. We cannot infer that these results are due to a reduction in cell viability. We postulate that this observation may be due to an increase in differentiation, as evidenced by collagen synthesis, MTT, and the synthesis of non-collagenous proteins and mineralization nodules, which are discussed below. The MTT assay is a cell viability test; the formation of formazan crystals depends on the mitochondrial activity of viable cells. However, mitochondrial activity also varies with cell maturity, and the decrease in mitochondrial activity during maturation means that differentiated cells frequently show a reduction in formazan crystal formation [49,50]. At caffeine doses of 25 mg/kg and 50 mg/kg, the osteoblasts showed decreased conversion of MTT into formazan crystals after 7 and 14 days of culture. At 21 days, the group treated with 100 mg/kg caffeine showed higher conversion of MTT into formazan crystals than the controls. Except for the 50 mg/kg group, it is difficult to explain the changes observed in the MTT assays of the groups that were treated with caffeine. Because the MTT assay is dependent on mitochondrial activity, more studies should be conducted to study the effects of caffeine on these cellular organelles.

Of the three doses of caffeine studied, the 50 mg/kg dose resulted in elevated type I collagen and alkaline phosphatase expression, enhanced synthesis and mineralization of nodules and increased expression of non-collagenous proteins such as osteocalcin, collagen, sialoprotein, Runx-2 and osteopontin compared with the control group at the studied time points. These proteins are important indicators of late osteogenic differentiation [41,51-57]. Therefore, it can be inferred that the osteoblasts cultured from the offspring of rats treated with caffeine at a dose of 50 mg/kg showed increased osteogenic potential in the early and late stages of differentiation. The increased osteogenic potential of osteoblasts in this study should be interpreted with caution, and further studies are needed to understand the relationship between these results and the bone changes observed *in vivo*. This increase should not necessarily be interpreted as beneficial for bone growth because any imbalance in cellular function, whether an increase or decrease, may impair skeletogenesis. Caffeine binds to adenosine receptors, and modulate several others receptors including glucocorticoid receptors, insulin, estrogen, androgen, vitamin D, cannabinoid, glutamate and adrenergic receptors, all of which are expressed in osteoblasts or osteoprogenitor cells and have important functions during osteoblast differentiation [23,58-72]. However, little is known about the action of caffeine on these receptors or the resultant effects on endochondral bone formation and growth. Therefore, unfortunately, it is too early to determine the implications for humans or to suggest a mechanism of action for the observed increase in osteogenic factors. Numerous factors in the cellular microenvironment could also be responsible for the observed effects, although more studies are needed to identify these factors and elucidate their mechanisms of action.

Although 25 mg/kg of caffeine also increased the expression of osteocalcin, sialoprotein and osteopontin at two of the time points studied, the synthesis of mineralization nodules was not affected at this dose. This result is likely due to the reduction of collagen expression observed in this group. The formation of mineralization nodules depends on the synthesis of non-collagenous proteins and collagen prior to the mineralization process [41]. A dose of 100 mg/kg caffeine also promoted changes in the transcript levels of osteocalcin, osteopontin and sialoprotein at 7 days but did not alter the synthesis of mineralization nodules compared with the controls. However, it is important to note that with the exception of the MTT assay at 21 days, the 25 mg/kg and 100 mg/kg doses were similar to the controls in all parameters evaluated.

## Conclusions

In conclusion, osteoblasts extracted from the pups of rats that received caffeine at a dose of 50 mg/kg during pregnancy exhibit increased expression of osteocalcin, osteopontin, sialoprotein, runx-2, alkaline phosphatase, and type I collagen, resulting in increased synthesis of mineralization nodules. Our work presents novel results using methodology not previously performed and demonstrates the need for further studies to better understand the significance of our results and the differences in effects on osteogenic potential when caffeine is administered *in vivo* or directly to culture media.
